# Impact of social and mobility restrictions in Parkinson’s disease during COVID-19 lockdown

**DOI:** 10.1186/s12883-021-02364-9

**Published:** 2021-08-30

**Authors:** Raquel Luis-Martínez, Roberto Di Marco, Luca Weis, Valeria Cianci, Francesca Pistonesi, Alfonc Baba, Miryam Carecchio, Roberta Biundo, Chiara Tedesco, Stefano Masiero, Angelo Antonini

**Affiliations:** 1 Department of Neurosciences, University of the Basque Country, (UPV/EHU), Leioa, Spain; 2 Parkinson and Movement Disorders Unit, Department of Neurosciences, University of Padova, Via Giustiniani 5, 35138 Padua, Italy; 3 Rehabilitation Unit, Azienda Ospedaliera Universitaria di Padova, Padova, Italy; 4 Department of General Psychology, University of Padova, Padova, Italy; 5 Physical Medicine and Rehabilitation School, University of Padova, Padova, Italy

**Keywords:** Parkinson’s disease, COVID-19, Rehabilitation, Physical activity, COVID-19 Restrictions, Quarantine, Social isolation

## Abstract

**Background:**

The consequences of strict COVID-19 mobility restrictions on motor/non-motor features in Parkinson’s disease (PD) have not been systematically studied but worse mobility and quality of life have been reported. To elucidate this question, 12 mild to moderate PD patients were assessed in March 2020 before and after two months of isolation as part of a clinical study that had to be interrupted due to the pandemic and the implementation of COVID19 mobility restrictions.

**Methods:**

Twelve patients were systematically evaluated before and after the lockdown period as part of a larger cohort that previously underwent thermal water rehabilitation. Clinical outcomes were the Body Mass index, the Mini-Balance Evaluation Systems Test, the MDS-Unified Parkinson’s Disease Rating Scale part III, the 6 Minute Walking Test and the New Freezing of Gait Questionnaire. Global cognition was evaluated with the Montreal Cognitive Assessment scale. The impact of COVID-19 restrictions on quality of life and functional independence was evaluated with The Parkinson’s disease Quality of life (PDQ-39), the Activities of Daily Living (ADL) and Instrumental Activities of Daily Living questionnaires (IADL) and the Parkinson’s disease cognitive functional rating scales (PD-CFRS).

**Results:**

After two months of isolation the Mini-BESTest score worsened (*p*=0.005), and four patients reported one or more falls during the lockdown. BMI increased (*p*=0.031) while the remaining clinical variables including quality of life did not change.

**Conclusion:**

We observed moderate worsening at Mini-BESTest, greater risk of falls and increased body weight as consequence of prolonged immobility. We believe negative effects were partially softened since patients were in contact with our multidisciplinary team during the lockdown and had previously received training to respond to the needs of this emergency isolation. These findings highligh the importnace of patient-centered interventions in PD management.

## Background

The COVID-19 pandemic has been declared as a global emergency by the World Health Organization. Unprecedented restrictions have been implemented to control the viral spread, including a complete lockdown of quarantine isolation in certain countries including Italy [1]. Concerns about the severe restrictions in mobility and the subsequent sedentary lifestyle have been raised when dealing with chronic neurological conditions, such as Parkinson’s disease (PD) [2–5]. Falls are more common (up to 40–70% per year) in advanced PD and their frequency and severity increase with physical inactivity [6]. Overall, people with PD are twice more likely to experience falls than healthy elderly population [7]. Sedentary time was significantly related to several aspects of quality of life, including perceived deficits in mobility, cognitive processing (e.g., memory, concentration), and communication (e.g., difficulty with speech) [8]. Moreover, it is well-known that social deprivation affects cognition, mood and quality of life [9, 10].

Since, physical exercise programs are essential components in the management of motor and non-motor symptoms in PD patients [11–13], the main objective of this pre-post interventional study was to explore the effects of COVID-19 related social and physical restrictions on this population.

## Methods

### Participants and ethics statement

Twelve PD participants from our Movement Disorders and Parkinson Unit of the University Hospital of Padua (Italy) were tested before and after the 2-months lockdown period in Italy.

**Fig. 1:**
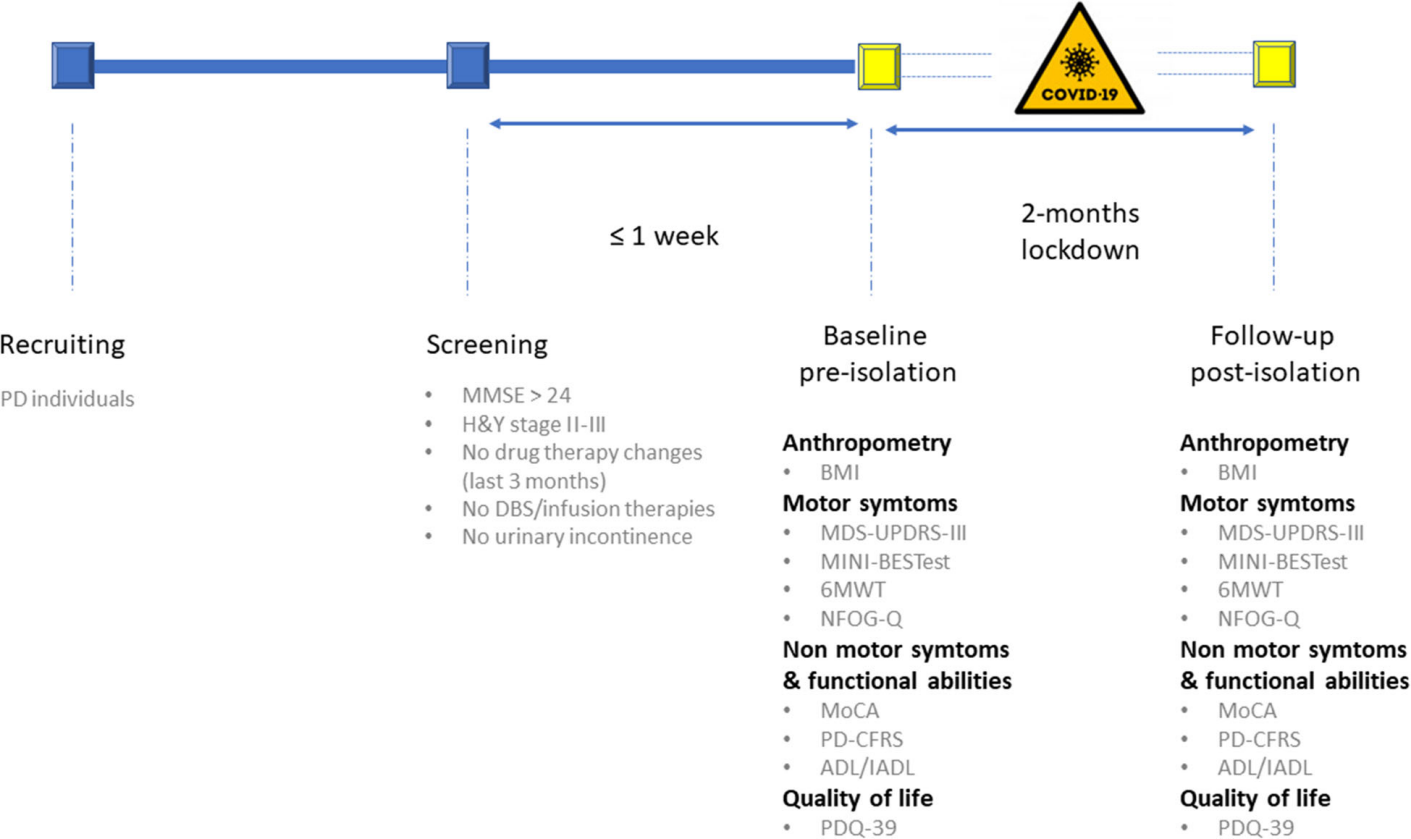
Pre- and post-isolation evaluation protocol

All participants had at screening (max 1 week before the baseline visit): (i) diagnosis of idiopathic PD [14]; (ii) Hoehn and Yahr (H&Y) score range 2-3 in the “OFF” state [15]; (iii) Mini Mental State Examination (MMSE) [16], score > 24; and (iv) stable pharmacological treatment for the last 3 months. We excluded participants who: (i) changed pharmacological treatment between baseline and follow-up visits (i.e., during the 8 weeks of lockdown); (ii) were demented based on MMSE score and iADL and ADL or unable to understand and sign the consent form; (iii) had deep brain stimulation and infusion therapies; (iv) suffered from diabetes; (v) reported pathologies of the musculoskeletal system; (vi) had history of brain stroke, myocardial infarction or suffered from uncontrolled hypertension; (vii) reported urinary incontinence considering that the rehabilitation program was planned in thermal water; and (viii) reported relevant brain comorbidities or cerebrovascular disease, as assessed with clinical T1w3D and FLAIR MRI protocol. All patients were part of a larger cohort that previously underwent thermal water rehabilitation. The whole group underwent an extensive evaluation assessing clinical, motor and cognitive aspects, which was administered two weeks prior (pre-isolation: before March 11, 2020) and following the lockdown in Italy (post-isolation: after May 4, 2020). The two evaluation sessions were carried out at the same day time during patients ON phase (see Fig. 1).

In order to be sure that participants had no close contact with potential COVID-positive people or became COVID-positive themselves between the two evaluation sessions, a triage questionnaire was administered both by telephone and in person at post-isolation visit. Namely it was checked whether i) their body temperature was lower than 37.5 °C, ii) they experienced COVID-19 symptoms; iii) their family members or close contacts (less than 1 meter in the same room for at least 15 min without protective equipment) confirmed or suspected to have contracted COVID-19 in the 20 days prior the evaluation; iv) they have ever been tested for COVID-19, especially in the 20 days prior the evaluation; and eventually if v) they have ever been positive to COVID-19 tests. None of the patients included in this study had history of positive COVID-19 or had contact with positive people.

Participants were evaluated in the context of a project that aimed to study the effect of rehabilitation in thermal water on people with PD. This rehabilitation program was interrupted due to the COVID-19 lockdown, but all clinical assessments at baseline were successfully performed the 2-weeks before lockdown. Participants were evaluated by the multidisciplinary team in our clinic, which includes a nurse case manager, neurologists, psychologists, physiotherapists and bioengineers working in collaboration with the rehabilitation unit and a study coordinator. Our approach to patients’ management includes education and training according to current recommendations [17, 18]. Assistance via phone calls and emails to those patients who asked for it was granted during the lockdown period and included training about low-intensity physical exercises aimed to relieve immobility-associated pain (i.e., stretching exercises for neck, trunk and lower limbs) and drugs administration. Although being of help, it is worth noting that this intervention is far from the concept of delivering a proper telemedicine where patients can actively interact with clinicians.

The study was carried out according to the Declaration of Helsinki and ethical approval to data collection was granted by the local review board. Participants read and signed an informed written consent form before participating to the study.

### Clinical and motor assessment

Clinical description (age at disease onset, disease duration, age, gender), and motor aspects (Unified Parkinson’s Disease Rating Scale part III (MDS-UPDRS III) [19] and Hoehn and Yahr (H&Y) Scale [15]) were collected by a neurologist experienced in the field of movement disorders. Levodopa (LEDD) and dopamine agonist equivalent daily (DAED) doses were calculated [20], as well as the presence of ongoing anticholinergic treatments.

A physical therapist specialized in movement disorders collected the participants’ body mass and height to calculate the participants’ body mass index (BMI, kg/m^2^) and carried out the motor evaluation. The Mini-Balance Evaluation Systems Test (Mini-BESTest), a shortened version of the Balance Evaluation Systems Test (BESTest), was employed as clinical tool to measure balance and predict the risk of falls [21]. The Mini-BESTest consists of 14 items (scored from zero to two) organized in four sections: anticipatory postural adjustments (APAs), reactive postural control (RPC), sensory orientation (SO) and dynamic gait (DG). The latter includes the Timed Up and Go (TUG) and the Timed Up and Go with Dual Task (TUG-DT). The TUG with and without dual task is used to determine the effects of cognitive load on gait performance. In the first three sections of the test, the maximum score is six and for the latter is 10, with a maximum total score of 28 points describing perfect motor conditions, as described by [21]. The Mini-BESTest is a significant predictor of falls and detector of balance impairment in PD [22, 23], with one being addressed as potential faller when the total score is ≤ 17.5/28 points [24].

Six Minute Walk Test (6MWT) was used to assess participants’ aerobic capacity and endurance. The distance covered over a time of six minutes is recorded as the outcome on which to contrast changes in performance and capacity. The test was carried out in an obstacle-free corridor with a length of 30 meters [25].

The New Freezing of Gait Questionnaire (NFOG-Q) is a self-reportable questionnaire consisting of nine items that measure presence, severity and relevant impact of freezing of gait (FOG) in individuals’ daily life [26]. The higher the score, the larger the impact of FOG in individuals’ daily life.

### Cognitive and functional assessment

A clinical semi-structured interview was carried out by two experienced neuropsychologists performing the cognitive-behavioral evaluation. The Montreal Cognitive Assessment scale (MoCA) was administered to evaluate global cognitive status as it has been shown that MoCA is a sensitive and clinically useful cognitive screening instrument in parkinsonisms [27–30]; The MoCA good sensitivity is likely to be due to the lack of ceiling effect, since it explores attention-executive function whose performance has been previously associated with nigrostriatal alterations [31, 32] and/or brain dopamine level [33]. The MoCA is an 8-sections and 30-points scale with short time of administration assessing visuospatial and executive functions, naming ability, memory, attention, language, abstraction, recall and orientation [34]. In order to avoid learning effect between evaluations, alternative MoCA versions were adopted at baseline and post-evaluation visit [35]. Further, as one of the common characteristic of cognitive impairment is the functional decline in instrumental activities of daily functioning and subjective cognitive complaints, we investigated their presence by using the Parkinson’s disease cognitive functional rating scale (PD-CFRS) [36]. Its performance allows to avoid motor biases in capturing the functional impact of cognitive impairment in Parkinson's disease and thus to adequately detain the clinical significance of cognitive change. A score below three is associated with normal cognition, a score above or equal to three is associated with mild cognitive impairment and a score above or equal to six to dementia. Moreover, we also administered the Activities of Daily Living (ADL) [37] and the Instrumental Activities of Daily Living (IADL) [38] questionnaires to further explore other aspects of daily life (such as occupational and personal care). The ADL score range from 0 to six and the total score below six is supportive of dementia. The IADL score range from 0 to 8 (from female) and 0 to 6 (from male) with 8/6 score means preserved functionality. Quality of life was evaluate administering the Parkinson’s disease Quality of Life Questionnaire (PDQ-39), which is a 39-items questionnaire specific for PD, aiming to self-evaluate the impact of the disease in individuals’ quality of life in the following domains: (i) mobility, correlated with physical function (10 items); (ii) activities of daily living, associated with limitations due to physical problems (six items); (iii) emotional well-being, associated with mental health (six items); (iv) stigma (4 items); (v) social support, (3 items); (vi) cognition (four items); (vii) communication (four items); and (viii) bodily discomfort, associated with pain (three items) [39]. Each question is expected to be scored from zero (never) to five (always). The higher the PDQ-39 total score, the larger the impact of PD in individuals’ daily life. To ensure consistency of the results, the same neuropsychologist administered questionnaires and tests to each participant pre- and post-isolation.

### Statistical analysis

Statistics were calculated using IBM SPSS Statistics (version 19.0, SPSS Inc., Chicago, Illinois).

For each variable, Minimal Detectable Change (MDC) derived from literature was considered to evaluate the noticeable change in ability [40]: Mini-BESTest (MDC=4.1 [41]); MDS-UPDRS part III (MDC=4.63 [42]), 6MWD (MDC=82 m [43]), NFOG-Q (MDC=3 [44]), BMI (MDC=2 kg/m^2^ [45]), MoCA (MDC=3 [46]).

The normality of scores distributions was assessed using the Shapiro-Wilk test. Pre-post lockdown effects on continuous variable were assessed with paired Two-tailed Student’s t-tests and on categorical variables with McNemar test. For those variables that do not satisfy normality distribution, Median, 25-75-percentile values and the paired Wilcoxon signed-rank test were considered. Effect size power analysis was calculated using Cohen’s *d*_*Z*_ measure correcting for small sample size and between-repetitions Pearson correlation [47]. Achieved power was calculated using G*power 3.1.9.4 tool [48]. An achieved power 0.8 and alpha =0.2 were considered significant for the power analysis. Significance was set at p < 0.05 (two-tailed). In order to test the dependency of pre-post effect from baseline motor status, a GLM ANCOVA was run between variables’ delta change and baseline MDS-UPDRS part III score.

## Results

Participants’ sociodemographic and clinical characteristics at the screening visit (pre-isolation period) are shown in Table 1. Throughout the whole quarantine period, there were no changes in drug therapy. Four individuals reported more than one fall during this period, whereas only one reported falls prior the baseline visit. Six participants reported drug-controlled hypertension, and two of them had suffered from ischemic heart disease. None of our participants was on acetylcholinesterase inhibitors (AChE).

**Table 1:** Social and demographical patients’ characteristic at the screening visit. Data are given as median values with 1^st^ and 3^rd^ quartiles (25- and 75-percentile, respectively Q1 and Q3), or as frequencies (N, %)

	Whole PD sample (*n*=12)
	Median (Q1; Q3) /Frequency
Age (years)	69.5 (67.0; 73.8)
Gender (male)	8 (67%)
Education (years)	10.5 (8.0; 13.8)
Age at disease onset (years)	58.0 (54.0; 64.25)
Disease duration (years)	10.0 (8.0; 13.3)
LEDD (mg/die)	745.0 (590.0; 1298.5)
DAED (mg/die)	105.0 (45.5; 210.0)
H&Y	2.5 (2; 3)
MMSE (corrected score)	29.0 (29.0; 29.3)
Live alone	2 (16%)
Freezing of Gait	7/5 (59%)
Tremor	9/3 (75%)

### Impact of lockdown on clinical and motor performance

Ten participants asked for telephone assistance during the lockdown period as they reported pain associated with poor daily mobility. The other two participants reported a good autonomy in self delivering stretching exercises that were performed on a daily basis.

Table 2 shows the results obtained for each variable pre- and post-isolation, as well as the relevant statistical results. A significant increment in the BMI was detected (*p* = 0.031, *d*_*z*_ = 0.703). Differences in MDS-UPDRS part III total score were not significant (*p* = 0.092). Six subjects showed an increasing of the MDS-UPDRS part III equal or superior to +4.63 points. Four of them remained stable and the other two showed a variation of -3.25 points. The Mini-BESTest total score was significantly reduced (*p* = 0.005, *d*_*z*_ = 0.994). Namely, an increased number of participants (six at the post-isolation vs. one at pre-isolation visit) obtained less than 17.5 points, the PD cutoff for potential fallers at the Mini-BESTest total score. In particular, four participants displayed a decline in the APAs sub-score; four participants worsened in the RPC, whereas similar values were observed in the SO and DG sub-scores in pre- and post-isolation visits. Post-hoc power analysis indicated that the power to detect obtained effects at the 0.2 level was 0.861 for the effect of lockdown on Mini-BESTest performance.

**Table 2:** Comparison of results of each variable pre- and post-isolation period. Data are given as median values with 1st and 3rd quartiles (25- and 75-percentile, respectively Q1 and Q3)

	Pre-isolation Median (Q1; Q3)	Post-isolation Median (Q1; Q3)	Cohen d_**z**_	Achieved Power^c^	*p*-value^a,b^
**BMI (kg/m**^**2**^**)**	26.5 (23.0; 29.6)	27.4 (23.2; 29.6)	0.703	0.841	0.031^bd^
**MDS-UPDRS Part III**	14.5 (11.8; 21.3)	19 (12.8; 31.0)	0.513	0.651	0.092^a^
**Mini-BESTest**	23 (21.0; 25.5)	19 (16.3; 21.0)	0.994	0.974	0.005^bd^
**6MWT (m)**	565.5 (370.8; 660.6)	510.0 (405.0; 665.0)	0.027	0.201	0.799^b^
**NFOG-Q**	6.0 (0.0; 13.8)	8.5 (0.0; 15.5)	0.353	0.454	0.313^a^
**MoCA** (corrected score)	24.2 (21.7; 26.0)	24.5 (23.3; 26.0)	0.257	0.345	0.392^b^
**PD-CFRS**	0.5 (0.0; 1.0)	1.0 (0.8; 1.0)	0.677	0.821	0.123^a^
**IADL**	5 (5.0; 6.3)	5 (5.0; 6.3)			
**ADL**	6.0 (6.0; 6.0)	6.0 (6.0; 6.0)			
**PDQ-39**	30.5 (17.0; 45.3)	32.5 (20.0; 44.3)	0.215	0.304	0.472^b^

Note. ^a^Wilcoxon signed-rank test or ^b^t-test for continuous variable and McNemar test for discrete variable. MDS-UPDRS Part III = MDS-Unified Parkinson Disease Rating Scale - part III; Mini-BESTest = Mini-Balance Evaluation Systems Test. 6MWT = Six-minute walk test; NFOG-Q = New Freezing of Gait Questionnaire. MoCA = Montreal Cognitive Assessment scale; PD-CFRS = Parkinson’s disease cognitive functional rating scale; ADL = Activities of Daily Living questionnaire; IADL = Instrumental Activities of Daily Living questionnaire; BMI = Body Mass Index; PDQ-39 = Parkinson’s disease Quality of Life Questionnaire. ^c^Post-hoc power analysis. Achieved power was calculated based on Cohen’s *d*_*z*_ and an α error probability of 0.2

^d^Significant isolation period effect (pre vs. post-isolation evaluation)

Additionally, statistical analysis revealed no significant differences in the 6MWT (*p*=0.799) and the NFOG-Q questionnaire (*p*=0.313) between pre- and post-isolation evaluations.

### Impact of lockdown restrictions on cognitive performance and quality of life

No differences were found in the ADL and IADL questionnaires. No significant effect was found on global cognitive performance as assessed with MoCA scale (*p*=0.392), nor in the PD-CFRS total score (*p*=0.123). Qualitative analysis of the PD-CFRS sub-items showed that four patients reported language (anomia/comprehension) problems and inability to remember drug intakes. Overall, PDQ-39 score did not change (*p*=0.472) but qualitative analysis shows a worsening in the "emotional well-being" domain in two subjects while 10 was in a similar condition.

## Discussion

To the best of our knowledge, this is the first study assessing the consequences of the COVID-19 related restrictions on both motor and non-motor symptoms of people with PD who had never contracted COVID-19. Considering that our subjects had been assessed just before the implementation of strict lockdown, we had the unique opportunity to test the impact of isolation without any treatment change and through a systematic and complete evaluation.

Our findings showed that no significant worsening in clinical measures and quality of life were detected for this cohort of PD patients in the mild and moderate stages of the disease. These findings should be interpreted considering that PD participants were followed regularly at our clinic and attended several educational activities at our local Parkinson lay association. We suggest that these results further point to the importance of multidisciplinary care interventions for PD patients to hand and learn self-management strategies [17, 18].

The only worsening item we detected was the Mini-BESTest total score. This finding could indicate that, if the lockdown period had continued, we could have possibly observed a clinically significant deterioration in balance performance of these individuals. The Mini-BESTest has proven to be a significant predictor of falls and detector of balance problems in PD [22, 23], and our result indicate that drastic mobility restrictions have a negative impact on balance. Indeed, the clinical cutoff to consider an individual as a faller via the Mini-BESTest is a total score equal to 17.5/28 points [24]. Contrarily to what observed before the lockdown (only 1 participant with Mini-BESTest total score < 17.5, who reported falls prior the baseline visit), six out of 12 participants did not pass such a threshold at the post-isolation visit, with four of them reporting falls during the 2-months lockdown. Four of them showed a decline in the APAs Mini-BESTest sub-items. Anticipatory Postural Adjustments (APA) are the necessary weight shift mechanism that occur prior to an internal or external postural challenge, such as step initiation and postural transfers (e.g. sit-to-stand task). Thus, this neural processing is required to achieve good control of center of mass transition in order to avoid a loss of balance and a subsequent fall [49, 50]. Remarkably, the rest of participants did not show clinical changes.

MDS-UPDRS motor examination total score highlighted only a modest median worsening. However, considering the minimal threshold of +4.63 points for detecting clinically meaningful worsening changes in the score of the MDS-UPDRS part III, this variation was clinically relevant for six of these participants [42]. The fact that only half of these participants showed a clinically negative effect, and that the 6MWT did not differ, could be explained with these participants and their caregivers having received an extensive training and education strategies for managing PD symptoms over the years at our clinic. Moreover, our multidisciplinary interventions were not substantially interrupted during this period thanks to remote-technology solutions. We succeeded in driving technology-based assistance for any requirement and promoting physical activity solutions in any case. This is consistent with the results of a recent survey for an Italian PD patients’ cohort about how the lockdown impacted on physical activity. This resulted in successful self-management patient’s strategies to continue physical activity at home. Moreover, education and awareness on the importance of physical activity were identified as main factors for the preservation of health in PD patients [51]. The body mass index experienced an increase, suggesting that this profound change in daily routine and regular physical activity had resulted in increased body weight, although this may not be specific of PD.

Finally, it should be noticed that baseline clinical, cognitive and functional status was relatively mild for this cohort and PDQ-39 score confirmed that patients were stable, and symptoms were well controlled with pharmacological treatment. Emotional wellbeing was the only domain showing a trend for worsening most likely due to prolonged social isolation [52]. Overall, it should be considered the relative shortness of the isolation period (8 weeks) together with the good baseline values of these patients and the uninterrupted remote assistance have possibly softened negative impacts of strict lockdown.

### Limitations of the study

The present study has two main drawbacks. The unprecedented conditions of this pandemic gave us the unique opportunity to explore the impact of a full lockdown on motor and non-motor symptoms in PD patients, but also limited the possibility to both perform an a-priori sample size calculation for our population, and to recruit an aged-matched control group. It is also worth noting that seven of the 12 participants completed an intensive motor rehabilitation program before the lockdown period, thus the overall outcome of the results might be affected. All the participants were yet at similar stage of the disease at the baseline. Another limitation worth mentioning is the unavoidable lack of control on how each participant managed to achieve a good quality of either the suggested or self-administered low-intensity physical exercises during the lockdown period.

## Conclusion

Our study provides the unique opportunity to test, with the same clinical measurements, in real life and in unprecedented mobility restrictions, the effects of isolation in PD. Even if the overall changes were modest, we observed an increased risk of falls in selected individuals which is nonetheless potentially relevant. The potentially negative effects of lockdown may have been softened by previous participation of these patients to educational activities and continued availability of our multidisciplinary team.

## Data Availability

The data that support the findings of this study are available from the corresponding author, upon reasonable request.

## Abbreviations

PD: Parkinson’s disease
BMI: Body mass index
Mini-BESTest: the Mini-Balance Evaluation Systems Test
BESTest: the Balance Evaluation Systems Test
APAs: Anticipatory postural adjustments
RPC: Reactive postural control
SO: Sensory orientation
DG: Dynamic gait
TUG: the Timed Up and Go test
TUG-DT: the Timed Up and Go Dual Task test
MDS-UPDRS-III: the MDS-Unified Parkinson’s Disease Rating Scale part III
6MWT: the 6 Minute Walk Test
NFOG-Q: the New Freezing of Gait Questionnaire
MoCA: The Montreal Cognitive Assessment
PD-CFRS: Parkinson’s disease cognitive functional rating scale
PDQ-39: the Parkinson’s disease Quality of life questionnaire
ADL: the Activities of Daily Living questionnaire
IADL: Instrumental Activities of Daily Living questionnaire
FOG: Freezing of gait
H&Y: Hoehn and Yahr Scale
MMSE: Mini-Mental State Examination
IQR: Interquartile range
